# Supplementary material for: Deep Learning for the Prediction of the Survival of Midline Diffuse Glioma with an H3K27M Alteration
Source: Brain Sci. 2023 Oct 19;13(10):1483. doi: 10.3390/brainsci13101483 (PMC10605651; doi:10.3390/brainsci13101483)
Supplement: Supplementary file 1 [file brainsci-13-01483-s001.zip › Tabel S1.pdf]

Table S1. The comparison of clinical information between the two groups.

Variable	Training set n=113	Test set N=23	P value
Age (Mean ± SD)	23.04±16.84	34.39±17.27	0.004[a]
Gender			0.222[a]
Male	53(46.9%)	14(60.9%)	
Female	60(53.1%)	9(39.1%)	
Tumor size			0.796[a]
≥1mm	11(9.7%)	1(4.4%)	
≥2mm	13(11.5%)	3(13.0%)	
≥3mm	53(46.9%)	10(43.5%)	
≥4mm	36(31.9%)	9(39.1%)	
Tumor location			0.409[a]
Thalamus	46(40.7%)	14(60.9%)	
Midbrain	4(3.5%)	1(4.3%)	
Pontine	42(37.2%)	5(21.7%)	
Medulla	3(2.7%)	1(4.3%)	
Basal ganglia	18(15.9%)	2(8.7%)	
Extent of resection			0.125[a]
GTR	21(18.6%)	9(39.1%)	
STR	28(24.8%)	4(17.4%)	
PR	54(47.8%)	7(30.4%)	
Biopsy	10(8.8%)	3(13.1%)	
Pre-op KPS (Mean ± SD)	71.15±17.10	74.35±15.62	0.409
Enhancement			0.637[a]
Yes	65(57.5%)	12(52.2%)	
No	48(42.5%)	11(47.8%)	
Radiotherapy			0.020[a]
Yes	31(27.4%)	12(52.2%)	
No	82(72.6%)	11(47.8%)	
Chemotherapy			0.303[a]
Yes	32(28.3%)	9(39.1%)	
No	81(71.7%)	14(60.9%)	
ATRX expression			0.022[b]
Yes	77(68.1%)	12(52.1%)	
No	26(23.0%)	11(47.9%)	
Missing	10(8.9%)	0	
P53 positive			1.000[b]
Yes	80(70.8%)	19(82.6%)	

No	17(15.0%)	4(17.4%)	
Missing	16(14.2%)	0	
Ki67 expression (Mean ± SD)	0.25±0.15	0.20±0.14	0.240
MGMT promoter methylation			0.817[b]
Methylated	20(17.7%)	6(26.1%)	
Unmethylated	64(56.7%)	17(73.9%)	
Missing	29(25.6%)	0	
Status			0.877[a]
Dead	102(90.3%)	21(91.3%)	
Alive	11(9.7%)	2(8.7%)	
Survival time (Mean ± SD)	9.41±12.11	10.37±8.78	0.719

[a] is for Chi-square test, [b] is for Fisher's exact test.
