# Supplementary material for: Deep Learning for the Prediction of the Survival of Midline Diffuse Glioma with an H3K27M Alteration
Source: Brain Sci. 2023 Oct 19;13(10):1483. doi: 10.3390/brainsci13101483 (PMC10605651; doi:10.3390/brainsci13101483)
Supplement: Supplementary file 1 [file brainsci-13-01483-s001.zip › Tabel S2.pdf]

Table S2. Optimal hyperparameters of DeepSurv and N-MTLR models.

Parameter	DeepSurv	N-MTLR model
Neural Network structure<br>(Layer: activation; nodes)	1: Softsign; 27<br>2: CosReLU; 45	1: Atan; 84<br>2: LogSigmoid; 20
Optimizer	rmsprop	sgd
Batch Normalization	False	False
Learning Rate	0.00018	0.00062
Number of Epochs	2106	88
Dropout	0.4	0.2
L2 Regularization	0.0082	0.0064
L2 Smoothing Regularization		0.0023
Bins		52
