# Supplementary material for: Deep Learning for the Prediction of the Survival of Midline Diffuse Glioma with an H3K27M Alteration
Source: Brain Sci. 2023 Oct 19;13(10):1483. doi: 10.3390/brainsci13101483 (PMC10605651; doi:10.3390/brainsci13101483)
Supplement: Supplementary file 1 [file brainsci-13-01483-s001.zip › Tabel S3.pdf]

Table S3. Optimal hyperparameters of Random Survival Forest.

Parameter	Random Survival Forest Model
Trees	216
Max Features	sqrt
Min Node Size	80
Sample Size Percent	80%
Importance Mode	Normalized Permutation
