# Supplementary figures and images for: Deep Learning for the Prediction of the Survival of Midline Diffuse Glioma with an H3K27M Alteration
Source: Brain Sci. 2023 Oct 19;13(10):1483. doi: 10.3390/brainsci13101483 (PMC10605651; doi:10.3390/brainsci13101483)

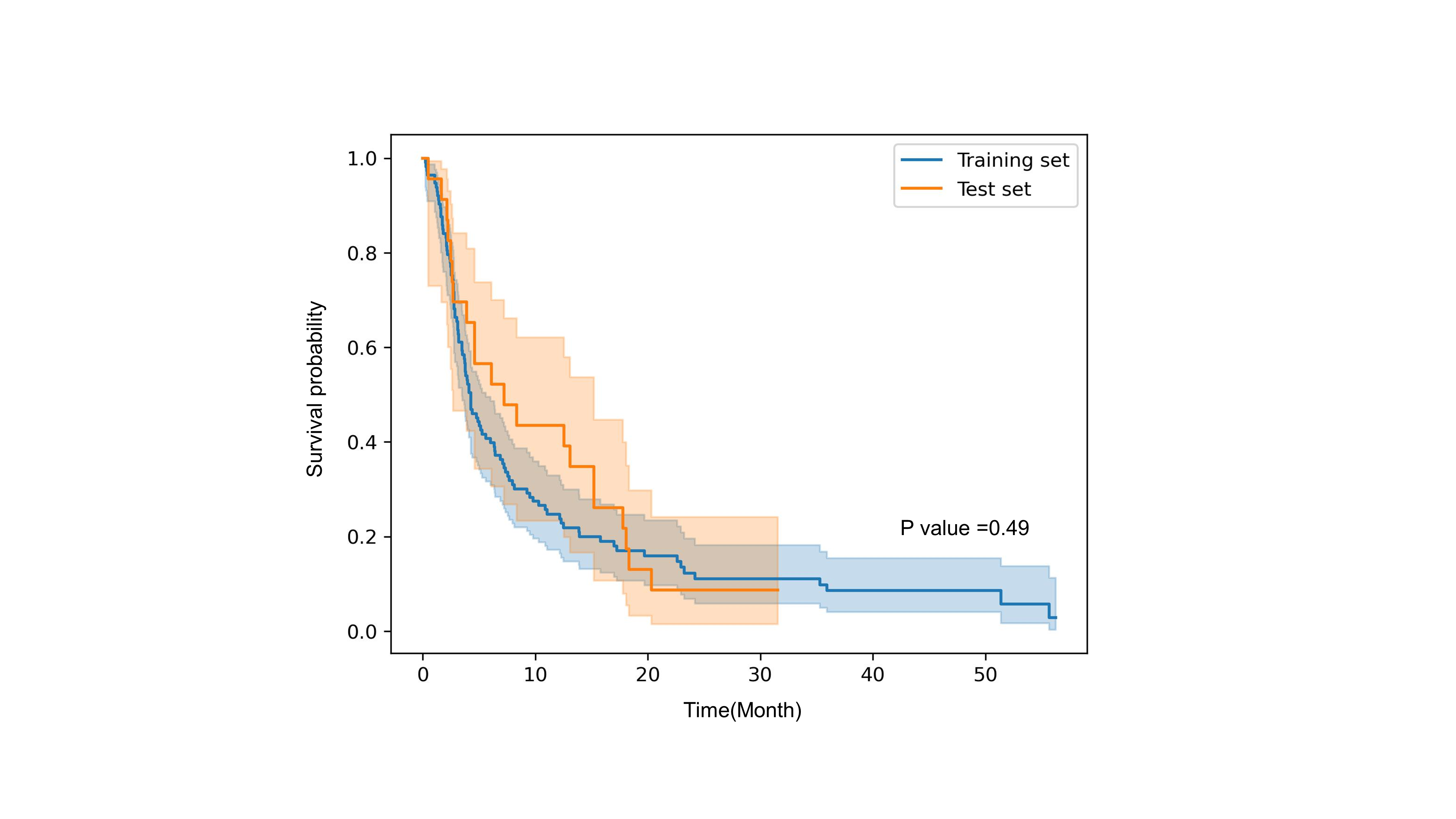

Supplement: Supplementary file 1 [file brainsci-13-01483-s001.zip › Figure S1.tif]

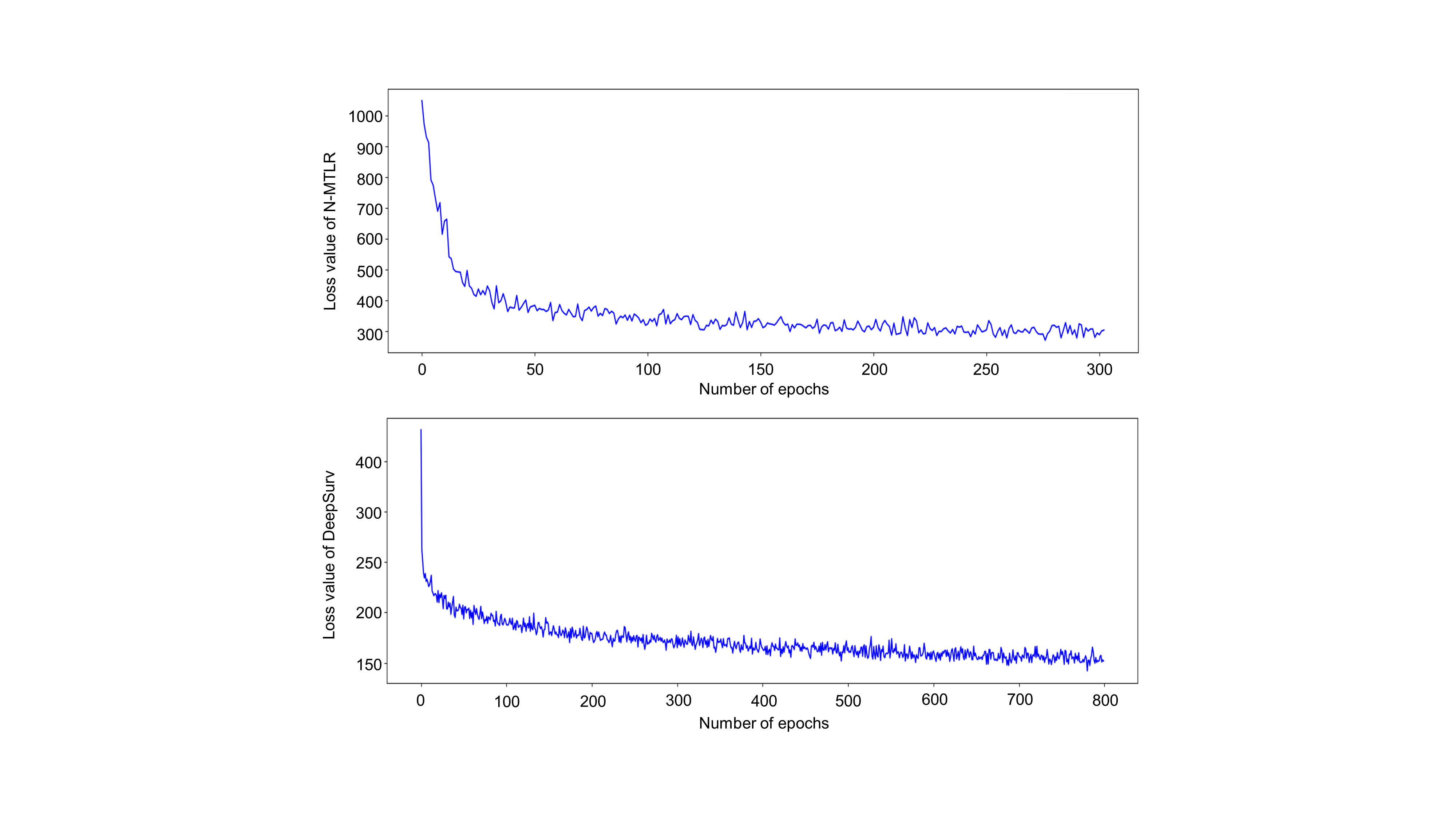

Supplement: Supplementary file 1 [file brainsci-13-01483-s001.zip › Figure S2.tif]

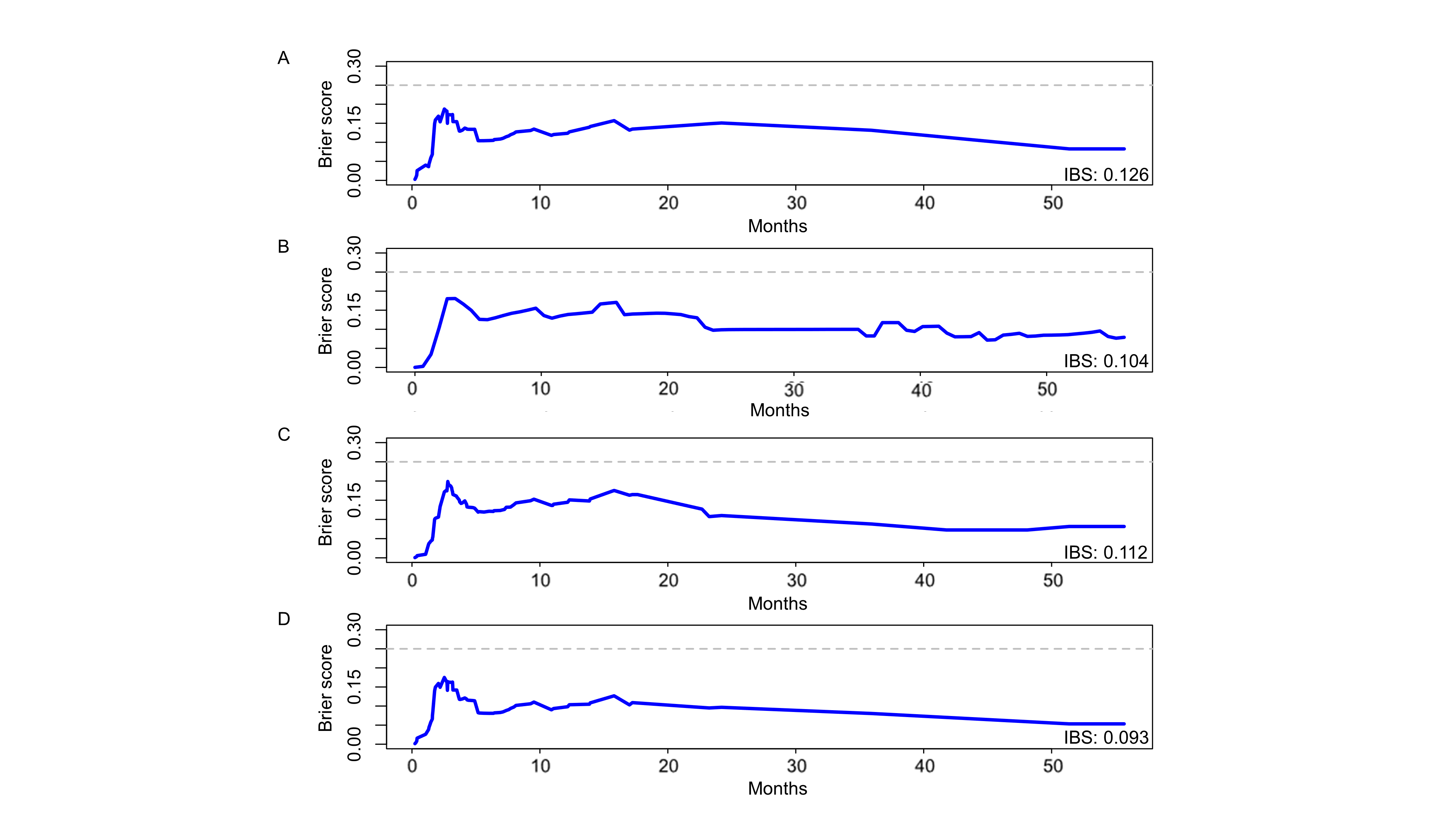

Supplement: Supplementary file 1 [file brainsci-13-01483-s001.zip › Figure S3.tif]

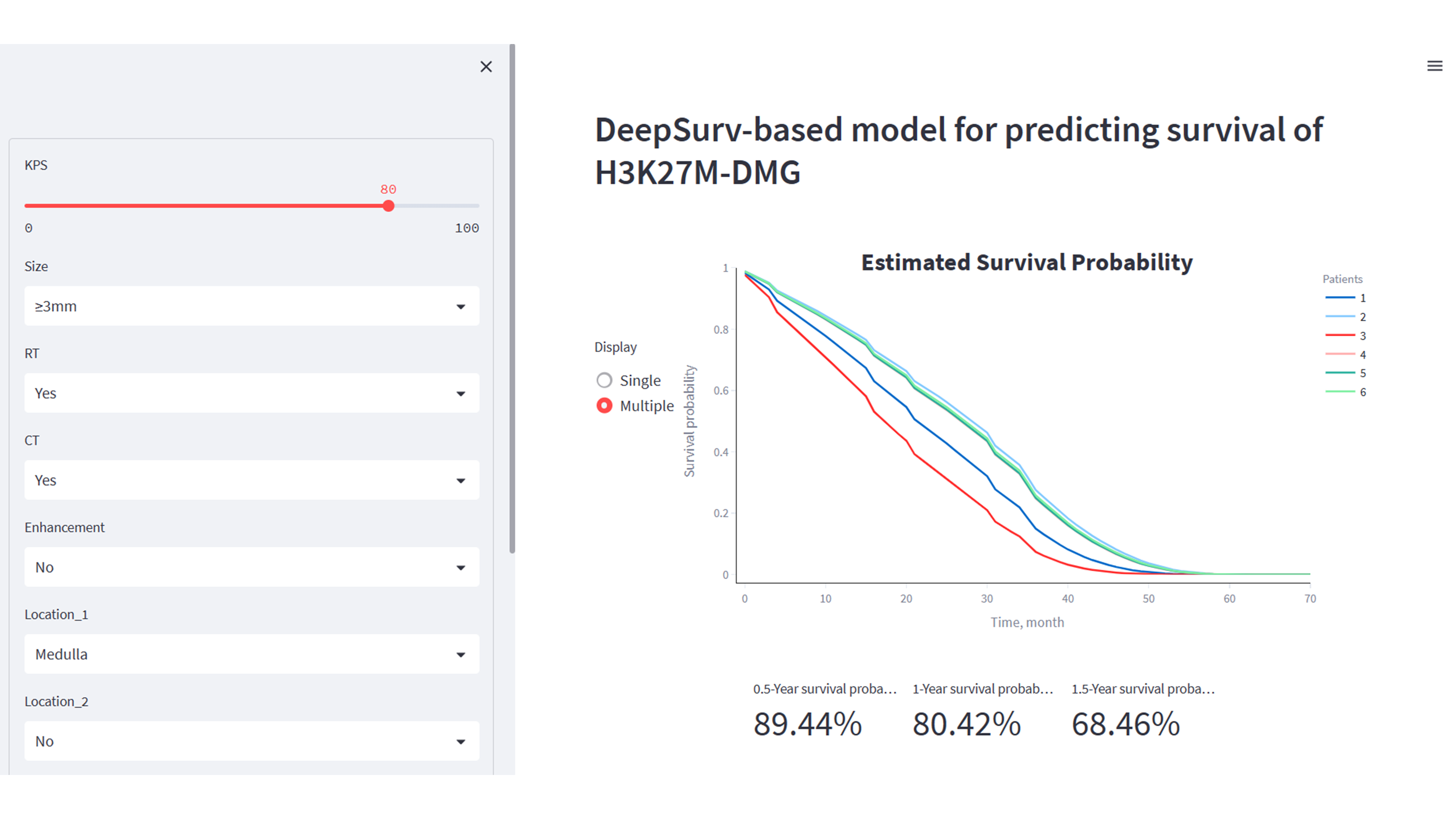

Supplement: Supplementary file 1 [file brainsci-13-01483-s001.zip › Figure S4.tif]
